# Supplementary material for: Dimensions of HIV-related stigma in rural communities in Kenya and Uganda at the start of a large HIV ‘test and treat’ trial
Source: PLoS One. 2021 May 17;16(5):e0249462. doi: 10.1371/journal.pone.0249462 (PMC8128261; doi:10.1371/journal.pone.0249462)
Supplement: S1 File — (DOCX) [file pone.0249462.s001.docx]

**SEARCH Trial Qualitative Cohort**

**BASELINE INTERVIEW – Year 1 of SEARCH**

Thank you for taking the time to talk with me today. I would like to ask you some questions about your experiences as a member of this community. I am interested in hearing about your experiences, opinions and beliefs in your own words. If there are any questions that make you feel uncomfortable, you should feel free to tell me and we can skip over those questions.

**Can you tell me about this community [***insert local place name***]?**

**Probes:** How long have you lived here? Were you born here? Were you married into this community? Did you migrate here from another community? Do you consider this community to be your home? How would you describe this community? If you could change anything about this community what would it be?

**What are some of the major problems that are faced by members of this community**?

**Probes:** How long have these problems existed? Have these always been a big problem, or did these problems come up recently? How do people in this community deal with these problems? What are some of the health problems faced by this community? If the participants do not mention HIV/AIDS as one of the major problems, probe specifically about this. Is HIV/AIDS a problem in this community? How are people in this community coping with the problem of HIV?

**How do people feel about HIV/AIDS in this community?**

**Probes:** Do you know people in your community who are infected with HIV or who have died of AIDS? How do you feel about these people? How are these people treated by others in this community? If someone you know told you that they were infected with HIV, how would you react? Why do some people fear to disclose their HIV status to their partner, family members, or others in the community? Do you talk to people about HIV/AIDS regularly? Can you tell me about the last conversation you had with someone when you talked about HIV? What specifically did you talk about? Why were you talking about this at that time?

**Antiretrovirals, called [site-specific term], are medications used to treat HIV/AIDS. They are medicines that are used to suppress or stop HIV, the virus that causes AIDS.** **Are you aware of drugs called antiretrovirals that are available to help treat people with HIV?**

**Probes:** If yes, tell me what you know about these drugs. How did you learn about these drugs? Do you know anyone in this community who is using these drugs? Where do they get the drugs that they need? Do people have to pay money to get these drugs? Do you have any concerns about drugs that are available to treat HIV? If yes, what are your concerns? How does the availability of antiretrovirals affect the way people in your community react to HIV infected people? How do people here view other people who are taking antiretrovirals?

**How would someone in this community learn their HIV status if they wanted to know?**

**Probes:** Do you ever talk to anyone about tests that can be done to learn your HIV status? Where do most people in this community go to take the test? Why do you think people would want to get their blood tested for HIV? Do you think that the availability of drugs called antiretrovirals to treat HIV might influence whether a person is willing to test for HIV? Why do you think people might not want to get their blood tested for HIV? If this test were available in your community, would you be interested in taking this test? Have you ever tested for HIV?

**If participant responds that he or she has previously tested for HIV:**

**Tell me about your HIV testing experience.**

**Probes:** When did you test? Why did you test? Where did you test? How did you feel about your testing experience? Did you talk to anyone about your decision to test before you got tested? Have you told others that you have tested for HIV? If yes, whom have you told? If not, why not? How did people react when you told them you tested?

**Can you tell me about your experiences sharing your HIV test results with other people after you were tested?**

**Probes:** Have you shared your HIV test results with anyone? If yes, whom? Did you share with your husband/wife/partner? Other family members? If yes, tell me about this experience – tell me about your decision to share your test results; tell me about their reaction when you shared your test results. Is there anyone you want to know your results that you have not told yet? If you did not share your results with anyone, why not? How has the availability of drugs called antiretrovirals to treat HIV affected your decision about sharing test results with people? Is there anyone who you do not want to know your HIV test results?

**If participant discloses that he/she is HIV seropositive:**

**Probes:** Can you please tell me, have you ever disclosed your HIV-positive status to anyone? If yes, who was the first person you disclosed to? Why did you choose that person? Were there others you wanted to disclose your status to, but didn’t feel you could? Can you tell me more about that? If no, can you tell me more about why you haven’t felt able to disclose your status to anyone?

**Have you ever received any services and/or treatment for HIV disease?**

**If the participant responds that he or she has NOT previously received care for HIV:**

**Can you tell me the main reasons why you have not yet received HIV care?**

**Probes**: Are there any other problems or issues that have kept you from getting services at the clinic for HIV? Please tell me, what would make it easier for you to enroll in HIV care and treatment?

**If the participant responds that he or she has previously received care for HIV:**

**Are you currently receiving regular HIV care?**

**Probes:** How has receiving HIV care/treatment affected your life? Where do you go for medical care? Probe for all locations. Why did you decide to seek care at that site? Have you ever had to change where you access HIV care at any point since you tested positive? If yes, probe for main reasons why. If not currently enrolled in care, can you tell me the main reasons you’re not receiving care currently? Probe for ALL reasons, including mobility, negative provider experiences, switched to traditional medicine, etc. Please tell me, what would make it easier for you to enroll again in HIV care and treatment?

**Have you ever missed HIV care appointments, or dropped out of care for a time?**

**Probes:** What were the circumstances that led this to happen? For about how long was your care interrupted? What are the main barriers you face, to being able to make appointments? Probe for ALL reasons for missing appts.: negative experiences with providers, issues of stigma and disclosure, problems with money or transportation, depression, other health issues, lack of someone else to take care of children, etc. Are there any other reasons? Please tell me, what would make it easier for you to make appointments?

**What are the main barriers you face to being able to adhere to taking your HIV medications?**

**Probes:** Probe for ALL reasons for missing medication doses.: negative experiences with providers, issues of stigma and disclosure, problems with money or transportation, need to hide medication-taking from others, depression, forgetfulness, denial, etc. Are there any other reasons? Please tell me, what would make it easier for you to adhere to taking your HIV medications?

**If the participant responds that he or she has NOT previously tested for HIV:**

**You mentioned to me that you have never been tested for HIV before. Can you tell me more about this.**

**Probes:** How do you feel about testing for HIV? What are some reasons you have not been tested for HIV before? Do you think you would ever want to be tested? If testing were available in your community, would you be interested in taking this test? Is there anything you think might change your mind about testing?

***Ask all participants, whether they have tested for HIV or not*.**

Now I would like to ask you some questions about your sexual relationships. If you are uncomfortable with any of these questions, you can skip those questions or stop the interview completely.

**Can you tell me about the partner you consider to be your primary relationship partner** (defined as a partner they have been with for at least 3-months and to whom they feel a special obligation or emotional attachment)?

**Probes**: How did you meet this partner? How long have you been with this partner? How often do you see this partner? Do you and your partner ever talk about HIV and your risk of HIV? If you do talk to your partner about HIV and HIV risk, can you tell me what you talk about? Do you feel that you are at risk for HIV because of this partner? If yes, tell me why you think you may be at risk. Please tell me about anything you are doing in your relationship to try to minimize this risk, or if you don’t feel able to do anything, please tell me about that. How has the availability of drugs called antiretrovirals to treat HIV affected this? Is there anything else you would like to do in your relationship to minimize your risk, yet you feel you are unable to do? If so, can you tell me why you feel like you are unable to do this with your primary partner?

**Can you tell me about your other current sexual relationships?**

**Probes**: How many other sexual partners do you currently have (in addition to your primary partner)? How long have you been with this/these partner(s)? Before this/these partner(s) when was the last partner you had? [If participant mentions more than one current partner], Are your partners aware that you have other sexual partners?

**SEARCH Trial Qualitative Cohort**

**SECOND INTERVIEW (Year 3 of SEARCH)**

**[Note prior to interview, ethnographer prepares a summary sheet with main findings from baseline interview. This is used to prepare individualized follow-up questions as needed.]**

Thank you again for taking the time to meet with me. Today, I would like to follow up with you on some of the questions we talked about 2 years ago, and then ask you some new questions. [It would be useful here for the ethnographer to remind the informant what they talked about in the last interview. This interview should build on what was described in this last interview. If there are points that need clarification, then those should also be asked in the beginning.]

**In the last interview we had you mentioned the following major problems faced by members of this community (recap what the participant told you in the baseline interview here). Do you still feel these are major problems faced by people in this community?**

**Probes:** Are there any other problems that you feel people now face? Have there been any changes in how people cope with these problems in the last two years? If the participants do not mention HIV/AIDS as one of the major problems, probe specifically about this. Is HIV/AIDS a problem in this community? If yes, how are people in this community coping with the problem of HIV?

**In the last interview we had you told me that people feel the following way about HIV in this community (recap what the participant told you in the baseline interview here). Do you think people feel the same way today about HIV/AIDS in this community?**

**Probes:** How has the way people feel about HIV/AIDS in this community changed during the last two years? Why do you think these changes have occurred? Since we last spoke have you known any other people in your community who are infected with HIV? How do you feel about these people? How are these people treated by others in this community? Have there been any changes in the way people with HIV are treated by others in this community? How has the availability of drugs called antiretrovirals for HIV affected this? If someone you know told you that they were infected with HIV, how would you react to them? Have you talked to anyone about HIV/AIDS in your community lately? If yes, can you tell me about one conversation about HIV/AIDS you have had with someone else in this community in the last 6 months. What specifically did you talk about? Why were you talking about this at that time?

**Since the time we last spoke, have you noticed any changes in your community related to attitudes and fears about HIV?**

**Probes:** What changes have you noticed? How did you notice these changes? Where did you notice these changes? Why do you think these changes occurred? Since we last spoke have you noticed any more discussion in your community related to HIV, testing, and treatment? Since the time we last spoke, has your attitude or have your fears about HIV changed? Why do you think these changes occurred?

**Since we last spoke are you aware of anyone in this community who is taking drugs called antiretrovirals to treat HIV?**

**Probes:** How did you learn they were taking these drugs? Where do they receive their drugs? Do you have any concerns about these drugs that are used to treat HIV? If yes, what are your concerns?

**Since we last spoke, have you known anyone in your community who has tested for HIV?**

**Probes:** Where did they test? How did you know that they tested? Did they tell you the results of their HIV test? How did you feel when they told you their test results? How did this person feel about their experience with testing? Do you think that the availability of drugs called antiretrovirals to treat HIV might influence whether a person is willing to test for HIV? Have you tested for HIV? If yes, when did you last test for HIV?

**If the participant responds that he or she has tested for HIV in the past year:**

**Can you tell me more about your testing experience?**

**Probes:** Why did you decide to test? Did you talk to anyone about your decision to test before you got tested? Where did you test? How did you feel about your testing experience? Did you feel that you got the support you needed during your counseling sessions? Did the counselor answer all of the questions? What would you have changed about the testing experience if you could change one thing? How did you feel learning your HIV test results at that time?

**After you were tested for HIV did you use any specific medical/health services?**

**Probes:** Tell me about the services you used. Where did you use these services? How did you learn about these services? How did you feel about these services? Are there any services you felt like you needed after you were tested, but you could not access?

**If participant discloses that he/she is HIV seropositive:**

**Can you tell me about your experiences sharing your HIV test results with other people after you were tested.**

**Probes:** After you were tested, did you talk to anyone about your experience with testing? Have you shared your HIV test results with anyone? If yes, whom? If yes, tell me about this experience – tell me about your decision to share your test results; tell me about their reaction when you shared your test results. How has the availability of drugs called antiretrovirals affected your decision about sharing test results with people? Is there anyone you want to know your results that you have not told yet? If you did not share your results with anyone, why not? Is there anyone whom you do not want to know your HIV test results? Can you tell me why you do not wish to disclose your test results to this person? What makes it difficult for you to share your results with this person? Did you share your HIV test results with your sexual partner? [If they mentioned that they had more than one sexual partner last time]: Did you share your HIV test results with your other sexual partners? Tell me about how you decided which partners to share your results with. How did these partner(s) react when you shared your results with them? How did it feel to discuss testing for HIV with your partner(s)? Can you tell me why you do not wish to disclose your test results to your partner(s) (or to a particular partner if more than one)? What makes it difficult for you to share your results with this/these partner(s)?

**Have you ever received any care for HIV disease?**

**If the participant responds that he or she has NOT previously received care for HIV:**

**Can you tell me the main reasons why you have not yet received HIV care?**

**Probes**: Are there any other problems or issues that have kept you from enrolling in care? Please tell me, what would make it easier for you to enroll in HIV care and treatment?

**If the participant responds that he or she has previously received care for HIV:**

**Are you currently receiving regular care?**

**Probes:** How has receiving HIV care affected your life? Where do you go for medical care? Probe for all locations. Have you ever had to change where you access HIV care at any point since you tested positive? If yes, probe for main reasons why. If not currently enrolled in care, can you tell me the main reasons you’re not receiving care currently? Probe for ALL reasons, including mobility, negative provider experiences, switched to traditional medicine, etc. Please tell me, what would make it easier for you to enroll again in HIV care and treatment?

**Have you ever missed HIV care appointments, or dropped out of care for a time?**

**Probes:** What were the circumstances that led this to happen? For about how long was your care interrupted? What are the main barriers you face, to being able to make appointments? Probe for ALL reasons for missing appts.: negative experiences with providers, issues of stigma and disclosure, problems with money or transportation, etc. Are there any other reasons? Please tell me, what would make it easier for you to make appointments?

**What are the main barriers you face to being able to adhere to taking your HIV medications?**

**Probes:** Probe for ALL reasons for missing appts.: negative experiences with providers, issues of stigma and disclosure, problems with money or transportation, etc. Are there any other reasons? Please tell me, what would make it easier for you to adhere to taking your HIV medications?

**If the participant responds that he or she tested for HIV prior to baseline, but has not re-tested since that time:**

**You mentioned that you tested for HIV once before the first interview we did together, but you have not been tested again since that time. Can you tell me more about your decision not to test again for HIV?**

**Probes:** Do you think there is any reason for a person to test more than one time for HIV? If yes, why have you decided not to test again for HIV? Do you think that you may decide to test again at some point? If testing were available in your community, do you think you would test again for HIV?

**If the participant responds that he or she has not tested for HIV:**

**You mentioned to me that you have never been tested for HIV before. Can you tell me more about this.**

**Probes:** How do you feel about taking an HIV test? What are some reasons you have not been tested for HIV before? Do you think you would ever want to be tested? If testing were available in your community, would you be interested in taking this test? Is there anything you think might change your mind about testing?

*Ask all participants, regardless of testing experience:*

**In the last interview we had together, you told me that you had _______ sexual partner(s). Are you with the same partner(s) that you were with before?**

**Probes:** Have you had any new sexual partners since we last talked? If you are no longer with your former partners, can you tell me why your relationships ended? In the last 6 months have you and your partner ever talked about HIV and your risk of HIV? If yes, can you tell me about the conversations you have had with your partner about HIV. Do you feel that you are at risk for HIV because of this/these partner(s)? If yes, tell me why you think you may be at risk. Do you do anything in your relationship to try to minimize this risk? How has the availability of drugs called antiretrovirals to treat HIV affected this? Is there anything else you would like to do in your relationship to minimize your risk, yet you feel you are unable to do? If so, can you tell me why you feel like you are unable to do this with your partner(s). If you have not talked to your partner(s) about HIV or your risk of HIV in the last year, can you tell me why it is difficult to talk to your partner about HIV? Have you talked to your partner about getting tested for HIV in the last year? If yes, can you tell me what you and your partner discussed. How did it feel to discuss testing with your partner? How does your partner feel about getting HIV tested? If you have not discussed HIV testing with your partner, can you tell me why it is difficult to discuss HIV testing with your partner?

**SEARCH Trial Qualitative Cohort**

**THIRD INTERVIEW (Year 5 of SEARCH)**

Thank you again for taking the time to meet with me. Today, I would like to follow up with you on some of the questions we talked about last time, and then ask you some new questions. [It would be useful here for the ethnographer to remind the informant what they talked about in the last interview. This interview should build on what was described in this last interview. If there are points that need clarification, then those should also be asked in the beginning.]

**In the previous interviews we had you mentioned the following major problems faced by members of this community (recap what the participant told you in the previous interviews here). Do you still feel these are major problems faced by people in this community?**

**Probes:** Are there any other problems that you feel people now face? Have there been any changes in how people cope with these problems in the last 5 years? If the participants do not mention HIV/AIDS as one of the major problems, probe specifically about this. Is HIV/AIDS a problem in this community? If yes, how are people in this community coping with the problem of HIV?

**In the previous interviews we had you told me that people feel the following way about HIV in this community (recap what the participant told you in the previous interviews here). Do you think people feel the same way today about HIV/AIDS in this community?**

**Probes:** How has way people feel about HIV/AIDS in this community changed during the last 2 years? Why do you think these changes have occurred? Since we last spoke have you known any other people in your community who are infected with HIV? How do you feel about these people? How are these people treated by others in this community? How has the availability of drugs called antiretrovirals for HIV treatment affected this? Have there been any changes in the way people with HIV are treated by others in this community? If someone you know told you that they were infected with HIV, how would you react to them? Have you talked to anyone about HIV/AIDS in your community in the last year? If yes, can you tell me about one conversation about HIV/AIDS you have had with someone else in this community in the last year. What specifically did you talk about? Why were you talking about this at that time?

**Since the time we last spoke, have you noticed any changes in your community related to attitudes and fears about HIV?**

**Probes:** What changes have you noticed? How did you notice these changes? Where did you notice these changes? Why do you think these changes occurred? Since we last spoke have you noticed any more discussion in your community related to HIV and HIV testing, specifically? Since the time we last spoke, has your attitude or have your fears about HIV changed? Why do you think these changes occurred?

**Since we last spoke are you aware of anyone in this community who is taking drugs to treat HIV?**

**Probes:** How did you learn they were taking these drugs? Where do they receive their drugs? Do you have any concerns about these drugs that are used to treat HIV? If yes, what are your concerns?

**Since we last spoke, have you known anyone in your community who has tested for HIV?**

**Probes:** Where did they test? How did you know that they tested? Did they tell you the results of their HIV test? How did you feel when they told you their test results? How did this person feel about their experience with testing? Do you think that the availability of drugs called antiretrovirals to treat HIV might influence whether a person is willing to test for HIV? Have you tested for HIV? If yes, when did you last test for HIV?

**If the participant responds that he or she has tested for HIV since the last interview:**

Thank you again for taking the time to meet with me. Today, I would like to follow up with you on some of the questions we talked about last time, and then ask you some new questions. [It would be useful here for the ethnographer to remind the informant what they talked about in the last interview. This interview should build on what was described in this last interview. If there are points that need clarification, then those should also be asked in the beginning.]

**In the last interview we had you mentioned the following major problems faced by members of this community (recap what the participant told you in the baseline interview here). Do you still feel these are major problems faced by people in this community?**

**Probes:** Are there any other problems that you feel people now face? Have there been any changes in how people cope with these problems in the last two years? If the participants do not mention HIV/AIDS as one of the major problems, probe specifically about this. Is HIV/AIDS a problem in this community? If yes, how are people in this community coping with the problem of HIV?

**In the last interview we had you told me that people feel the following way about HIV in this community (recap what the participant told you in the baseline interview here). Do you think people feel the same way today about HIV/AIDS in this community?**

**Probes:** How has the way people feel about HIV/AIDS in this community changed during the last two years? Why do you think these changes have occurred? Since we last spoke have you known any other people in your community who are infected with HIV? How do you feel about these people? How are these people treated by others in this community? Have there been any changes in the way people with HIV are treated by others in this community? How has the availability of drugs called antiretrovirals for HIV affected this? If someone you know told you that they were infected with HIV, how would you react to them? Have you talked to anyone about HIV/AIDS in your community lately? If yes, can you tell me about one conversation about HIV/AIDS you have had with someone else in this community in the last 6 months. What specifically did you talk about? Why were you talking about this at that time?

**Since the time we last spoke, have you noticed any changes in your community related to attitudes and fears about HIV?**

**Probes:** What changes have you noticed? How did you notice these changes? Where did you notice these changes? Why do you think these changes occurred? Since we last spoke have you noticed any more discussion in your community related to HIV, testing, and treatment? Since the time we last spoke, has your attitude or have your fears about HIV changed? Why do you think these changes occurred?

**Since we last spoke are you aware of anyone in this community who is taking drugs called antiretrovirals to treat HIV?**

**Probes:** How did you learn they were taking these drugs? Where do they receive their drugs? Do you have any concerns about these drugs that are used to treat HIV? If yes, what are your concerns?

**Since we last spoke, have you known anyone in your community who has tested for HIV?**

**Probes:** Where did they test? How did you know that they tested? Did they tell you the results of their HIV test? How did you feel when they told you their test results? How did this person feel about their experience with testing? Do you think that the availability of drugs called antiretrovirals to treat HIV might influence whether a person is willing to test for HIV? Have you tested for HIV? If yes, when did you last test for HIV?

**If the participant responds that he or she has tested for HIV in the past year:**

**Can you tell me more about your testing experience?**

**Probes:** Why did you decide to test? Did you talk to anyone about your decision to test before you got tested? Where did you test? How did you feel about your testing experience? Did you feel that you got the support you needed during your counseling sessions? Did the counselor answer all of the questions? What would you have changed about the testing experience if you could change one thing? How did you feel learning your HIV test results at that time?

**After you were tested for HIV did you use any specific medical/health services?**

**Probes:** Tell me about the services you used. Where did you use these services? How did you learn about these services? How did you feel about these services? Are there any services you felt like you needed after you were tested, but you could not access?

**If participant discloses that he/she is HIV seropositive:**

**Can you tell me about your experiences sharing your HIV test results with other people after you were tested.**

**Probes:** After you were tested, did you talk to anyone about your experience with testing? Have you shared your HIV test results with anyone? If yes, whom? If yes, tell me about this experience – tell me about your decision to share your test results; tell me about their reaction when you shared your test results. How has the availability of drugs called antiretrovirals affected your decision about sharing test results with people? Is there anyone you want to know your results that you have not told yet? If you did not share your results with anyone, why not? Is there anyone whom you do not want to know your HIV test results? Can you tell me why you do not wish to disclose your test results to this person? What makes it difficult for you to share your results with this person? Did you share your HIV test results with your sexual partner? [If they mentioned that they had more than one sexual partner last time]: Did you share your HIV test results with your other sexual partners? Tell me about how you decided which partners to share your results with. How did these partner(s) react when you shared your results with them? How did it feel to discuss testing for HIV with your partner(s)? Can you tell me why you do not wish to disclose your test results to your partner(s) (or to a particular partner if more than one)? What makes it difficult for you to share your results with this/these partner(s)?

**Have you ever received any care for HIV disease?**

**If the participant responds that he or she has NOT previously received care for HIV:**

**Can you tell me the main reasons why you have not yet received HIV care?**

**Probes**: Are there any other problems or issues that have kept you from enrolling in care? Please tell me, what would make it easier for you to enroll in HIV care and treatment?

**If the participant responds that he or she has previously received care for HIV:**

**Are you currently receiving regular care?**

**Probes:** How has receiving HIV care affected your life? Where do you go for medical care? Probe for all locations. Have you ever had to change where you access HIV care at any point since you tested positive? If yes, probe for main reasons why. If not currently enrolled in care, can you tell me the main reasons you’re not receiving care currently? Probe for ALL reasons, including mobility, negative provider experiences, switched to traditional medicine, etc. Please tell me, what would make it easier for you to enroll again in HIV care and treatment?

**Have you ever missed HIV care appointments, or dropped out of care for a time?**

**Probes:** What were the circumstances that led this to happen? For about how long was your care interrupted? What are the main barriers you face, to being able to make appointments? Probe for ALL reasons for missing appts.: negative experiences with providers, issues of stigma and disclosure, problems with money or transportation, etc. Are there any other reasons? Please tell me, what would make it easier for you to make appointments?

**What are the main barriers you face to being able to adhere to taking your HIV medications?**

**Probes:** Probe for ALL reasons for missing appts.: negative experiences with providers, issues of stigma and disclosure, problems with money or transportation, etc. Are there any other reasons? Please tell me, what would make it easier for you to adhere to taking your HIV medications?

**If the participant responds that he or she has not tested for HIV:**

**You mentioned to me that you have never been tested for HIV before. Can you tell me more about this.**

**Probes:** How do you feel about taking an HIV test? What are some reasons you have not been tested for HIV before? Do you think you would ever want to be tested? If testing were available in your community, would you be interested in taking this test? Is there anything you think might change your mind about testing?

*Ask all participants, regardless of testing experience:*

**In the last interview we had together, you told me that you had _______ sexual partner(s). Are you with the same partner(s) that you were with before?**

**Probes:** Have you had any new sexual partners since we last talked? If you are no longer with your former partners, can you tell me why your relationships ended? In the last 6 months have you and your partner ever talked about HIV and your risk of HIV? If yes, can you tell me about the conversations you have had with your partner about HIV. Do you feel that you are at risk for HIV because of this/these partner(s)? If yes, tell me why you think you may be at risk. Do you do anything in your relationship to try to minimize this risk? How has the availability of drugs called antiretrovirals to treat HIV affected this? Is there anything else you would like to do in your relationship to minimize your risk, yet you feel you are unable to do? If so, can you tell me why you feel like you are unable to do this with your partner(s). If you have not talked to your partner(s) about HIV or your risk of HIV in the last year, can you tell me why it is difficult to talk to your partner about HIV? Have you talked to your partner about getting tested for HIV in the last year? If yes, can you tell me what you and your partner discussed. How did it feel to discuss testing with your partner? How does your partner feel about getting HIV tested? If you have not discussed HIV testing with your partner, can you tell me why it is difficult to discuss HIV testing with your partner?
