# Supplementary material for: Dimensions of HIV-related stigma in rural communities in Kenya and Uganda at the start of a large HIV ‘test and treat’ trial
Source: PLoS One. 2021 May 17;16(5):e0249462. doi: 10.1371/journal.pone.0249462 (PMC8128261; doi:10.1371/journal.pone.0249462)
Supplement: S2 File — (DOCX) [file pone.0249462.s002.docx]

**SEARCH Trial**

**BASELINE In-depth Interview Guide - Provider Qualitative Cohort**

*Thank you for doing this interview. We are here to discuss your experience with providing care to patients with HIV. We want to understand your experiences and your point of view, and are not here to criticize, so please feel free to share your real experiences with us.*

- What challenges do staff face in implementing streamlined ART delivery? What are the main challenges that providers perceive with regard to starting ART in patients with higher CD4 levels than national guidelines? Do these challenges differ by gender? How do the providers experience caring for patients who are sick when they start vs. mostly now healthy?
- What are providers’ perceptions of the effectiveness of enhanced services?
- What do providers perceive to be the main barriers to clients receiving services?
- What do providers see as the challenges in care in that patients present and are they medical, psychosocial, or logistical? And have these barriers changed over time?

**I. Experiences providing clinical care**

We want to learn a little bit about your work experiences providing care to HIV/AIDS patients.

- Why were you motivated to work in providing care and counseling for HIV infected patients?
- What formal training did you receive?
- How long have you worked at this particular clinic?

**II. Work setting**

Please tell us about the activities that make up your day to day routine work providing counseling, care and treatment to HIV-infected patients.

- About how many patients do you counsel about ART initiation or adherence each week?
- Do you provide counseling to individuals, couples or groups?
- Do patients tend to come alone, or with family members? Does this vary by gender and age?
- What aspects of your work do you find most frustrating or difficult? What aspects are the most rewarding?

**III. General ART initiation counseling content**

We would like you to tell us about your conversations with patients who were eligible for antiretroviral therapy either immediately before ART initiation or during the first weeks and months of ART to understand how you provide support and care.

- Which types of patients truly merit antiretroviral therapy, in your opinion?
- When discussing ART initiation with patients who are eligible for ART but not on ART already, what are the topics are the most important and most frequently discussed?
- Have these topics changed over time?
- What special issues do women face when discussing ART initiation? What about men?

For providers in intervention communities:

- What are the main challenges you have faced, so far, with implementing streamlined ART delivery? Are there certain types of patients that you’ve especially had difficulty enrolling for ART? (For instance, has your experience varied by health status, gender, age, or other characteristic of the patients?)
- What has been your experience so far with starting ART in patients with higher CD4 levels than the national guidelines? What has it been like to care for patients who are healthy when they start ART, versus sick when they start?

**IV. a. Patient-based factors in ART initiation and maintenance**

Now I’d like to ask you some questions about your experiences with patients, and the problems they have initiating ART and staying engaged in HIV care and treatment.

- First of all, what do most patients know about ART, before they initiate ART? What are the main misconceptions they have? How has this changed over time?
- What do you think most patients believe to be the benefits or drawbacks of ART? How has this changed over time?
- You mentioned that [recap what provider said about patients’ most common misconceptions or areas where they lack information]. How do you respond when patients have this misconception or need information? Can you please give me an example from among patients you have seen recently?
- Please tell me your opinion about the main differences between male and female patients in their knowledge, beliefs and attitudes about ART. Which are morely likely among men? Among women? What differences do you see among people in different age groups?
- What do you think people need to know about ART, in order to decide to start treatment? What other conditions need to be in place for people, in order for them to be able to initiate ART?
- What do you think most patients need to have or to believe in order to stay enrolled in care and treatment? And what do they need to have and to know in order to adhere to medications?

**IV. b. Clinic-based factors in ART initiation and maintenance**

We would like to learn more about the kinds of barriers that might exist at the clinic to ART initiation and engagement in care and treatment.

- From your point of view, what are the most important things that you see at the clinic, that might make it hard for patients to initiate ART? Please tell me about most people’s experience with waiting times. What are the interactions like with the staff who work here? How clearly are signs laid out, showing patients at the clinic where they should go?
- What do you think the clinic can do to make ART initiation easier for patients?
- What about staying engaged in care and treatment, specifically—what could the clinic do to help patients attend their regular appointments and adhere to medications?

**IV. c. Structural factors in ART initiation**

- Patients often have difficulty starting ART, because of problems due to lack of money, transportation, work or child care – What do you think are the biggest problems for patients in this clinic?
- Can you tell us if you have any suggestions or advice for patients who face problems with transportation, money or hunger?

**V. Wrap-up**

- Can you tell us about perhaps the best example of a difficult case you’ve had, with a patient was having problems starting ART, that that you were able to address? What did you provide to that patient (knowledge, advice or other support)?
- Can you tell us about one particularly difficult instance where the patient was unable to initiate ART despite having a clinical reason to start?
- Overall, many patients also get information about HIV care and treatment from community members as well as professional doctors and counselors. Is the information that your patients receive from elsewhere in the community sometimes different from the information provided at the clinic? If so, how does it differ? What or who are the other sources of information that community members turn to? In your opinion, do most people in the community trust the information they receive from the clinic, or from others in the community? How has this changed over time?
- What additional suggestions do you have for how providers can better help HIV-infected patients?

**SEARCH Trial Qualitative Provider Cohort**

**SECOND INTERVIEW (Year 3 of SEARCH)**

*[to be developed]*

**SEARCH Trial Qualitative Provider Cohort**

**THIRD INTERVIEW (Year 5 of SEARCH)**

*[to be developed]*
